# Relationships between enabling services use and access to care among adults with cardiometabolic risk factors: findings from the 2014 National Health Center Patient Survey

**DOI:** 10.1186/s12913-022-07739-3

**Published:** 2022-03-14

**Authors:** G. Sofia Martinez, Kellee White, Dahai Yue, Luisa Franzini, Craig S. Fryer, Ninet Sinaii, Dylan H. Roby

**Affiliations:** 1 Department of Health Policy and Management, University of Maryland, College Park School of Public Health, Room 3310, College Park, 20742 USA; 2 Department of Behavioral and Community Health, University of Maryland, College Park School of Public Health, College Park, USA; 3 Biostatistics and Clinical Epidemiology Service, Clinical Center, National Institutes of Health, Rockville, USA; 4 Department of Health, Society, and Behavior, University of California, Irvine, USA

**Keywords:** Enabling services, Cardiometabolic risk factors, Community health centers, Delayed care, Emergency room visits

## Abstract

**Background:**

Community health centers (CHCs) provide comprehensive primary and preventive care to medically underserved, low-income, and racially/ethnically diverse populations. CHCs also offer enabling services, non-clinical assistance to reduce barriers to healthcare due to unmet social and material needs, to improve access to healthcare and reduce health disparities. For patients with modifiable cardiometabolic risk factors, including obesity, hypertension, and diabetes, enabling services may provide additional support to improve disease management. However, little is known about the relationship between enabling services and healthcare accessibility and utilization among patients with cardiometabolic risk factors.

**Methods:**

This study uses data from the 2014 Health Center Patient Survey to examine the relationship between enabling services use and delayed/foregone care, routine check-ups, and emergency room visits, among adult community health center patients in the United States with cardiometabolic risk factors (*N* = 2358). Outcomes of enabling services users were compared to nonusers using doubly robust propensity score matching methods and generalized linear regression models.

**Results:**

Overall, enabling service users were 15.4 percentage points less likely to report delayed/foregone care and 29.4 percentage points more likely to report routine check-ups than nonusers. Enabling service users who lived in urban areas, younger and middle-aged adults, and those with two cardiometabolic risk factors were also less likely to report delayed/foregone care and/or more likely to report routine check-ups in comparison with nonusers. However, among adults with three or more cardiometabolic risk factors, enabling services use was associated with a 41.3 percentage point increase in emergency room visits and a 7.6 percentage point decrease in routine check-ups.

**Conclusions:**

The findings highlight the value in utilizing enabling services to improve timeliness and receipt of care among CHC patients with heightened cardiometabolic risk. There is a need for targeting high-risk populations with additional enabling services to support management of multiple chronic conditions.

**Supplementary Information:**

The online version contains supplementary material available at 10.1186/s12913-022-07739-3.

## Background

Community health centers (CHCs) play a large role in ensuring the availability of quality, comprehensive primary and preventive care to medically underserved, racially/ethnically diverse, low-income, and uninsured and under-insured populations throughout the United States [1–3]. Funded by the United States Department of Health and Human Services’ Health Resources and Services Administration (HRSA), approximately 13,000 CHCs serving almost 29 million individuals, operate across the country to mitigate health disparities through providing affordable community-based services [3]. As part of their comprehensive care, CHCs provide enabling services, defined by HRSA as “non-clinical services that do not include direct patient services that enable individuals to access health care and improve health outcomes” [4]^(p2)^. Enabling services encompass a wide range of services including assistance in making medical appointments, translation services, health education, and obtaining health insurance. These services may be useful for communities that are medically underserved, have unmet health-related social and material needs, and experience unique challenges to managing modifiable cardiometabolic risk factors.

Cardiometabolic risk factors (e.g., hypertension, high cholesterol, diabetes, and obesity) are major contributors to the leading causes of mortality in the United States [5–7]. Cardiometabolic risk factors have also been shown to be associated with higher health care expenditures due to increased hospital visits, pharmaceutical expenditures, and medical office visits [8, 9]. A disproportionate share of CHC patients report having at least one cardiometabolic risk factor [10]. The enabling services that CHCs provide, which are designed to facilitate greater access to care and provide support for unmet health-related social needs, may further assist patients with cardiometabolic risk factors effectively manage their conditions. However, empirical evidence supporting this assertion is limited and warrants further investigation.

Adults with cardiometabolic risk factors can face barriers to receiving and accessing care that may result in worse health outcomes. For example, those who are uninsured, under-insured, and receive public insurance (e.g., Medicaid coverage) are more likely to encounter challenges to paying for out-of-pocket expenditures related to medical care and prescription drugs [11, 12]. Additionally, for patients with cardiometabolic risk factors, receiving regular routine check-ups and assistance with making medical appointments, are important to allow providers time to offer interventions such as health behavior counseling and pharmacologic interventions that can improve outcomes and support management of cardiometabolic risk factors [13]. Enabling services may be beneficial in addressing these issues and improving disease management through ensuring timely access to care by providing assistance in arranging medical appointments and helping uninsured individuals enroll in health insurance.

Prior studies conducted among CHC patient populations, have shown improved cardiometabolic risk factor management with enabling service use utilization [14, 15]. For example, Weir et al. [15] demonstrated that greater enabling service use was associated with improved diabetes management. Additionally, it is possible that the provision of enabling services that address social determinants of health by providing material support for food, housing, income, and medication can lead to decreased severity and lower complications from cardiometabolic conditions [16]. However, methodological concerns with these studies related to smaller sample sizes, lack of robust approaches to account for selection bias, and narrow generalizability, limits the applicability of these findings to larger CHC populations. Although recent studies, using national samples of CHC patients demonstrate an association between enabling services use and greater health center visits, increased probability of having a routine check-up, and decreased likelihood of reporting the emergency department as a person’s usual source of care [17, 18], empirical research investigating enabling services and health care utilization among individuals with cardiometabolic risk factors is sparse.

To address these gaps in the literature, this study examined whether enabling services are associated with delayed/foregone medical care, routine check-ups, and emergency department (ED) visits among a nationally representative sample of CHC patients with at least one cardiometabolic risk factor using data from the Health Center Patient Survey. It is hypothesized that enabling services use will be associated with increased routine medical check-ups, and decreased delayed/foregone medical care and ED visits. Given prior research revealing heterogeneity in health care utilization outcomes by level of geography, age, and chronic disease burden [19–23], it is also hypothesized that the association between enabling service use and health care utilization will vary by geographic area (urban vs. rural), age, and burden of cardiometabolic risk factors. Identifying specific CHC patient population subgroups that benefit from utilizing enabling services to manage chronic conditions may provide additional critical evidence for increasing funding and budgetary support for CHCs to provide and finance these services.

## Methods

### Data source

The 2014 Health Center Patient Survey is a nationally-representative cross-sectional survey funded and administered by HRSA approximately every 5 years [24]. Briefly, the survey employs a three-stage sampling design to select the study population and selected patients are prescreened to ensure that they had at least one visit in the previous 12 months. In-person interviews were conducted in English, Spanish, Mandarin, Cantonese, Korean, and Vietnamese, by trained field interviewers generally onsite at the CHCs [25]. All of the variables, except for those on the location and type of health center are based on self-reported data from survey responses. Among the eligible patients, representing 169 health centers, 91% completed the survey, a total of 7002 respondents [25].

### Study population

We included study participants 18 years or older, who reported the health center as their usual source of care, visited the health center for at least a year, self-reported at least one cardiometabolic risk factor of diabetes, hypertension, high cholesterol, or a weight problem and did not have any missing data. Individuals were excluded from the sample if they were under 18 (20% of respondents), did not have any cardiometabolic risk factors (25% of respondents), had not visited the CHC as their usual source of care for at least 1 year (17% of respondents), or had missing data (5% of respondents) yielding a final analytic sample of 2358 adults.

### Dependent variables

Health care utilization was defined as reporting a recent routine checkup, delayed/foregone medical care, and ED visits. Survey respondents were asked “About how long has it been since your last general checkup or physical?,” with those reporting less than 12 months being coded as having had a recent checkup.

Delayed/foregone medical care was combined into one variable coded as one if an individual responded yes to the question “In the last 12 months, were you delayed in getting medical care, tests, or treatment you or a doctor believed necessary?” or the question “In the last 12 months, were you unable to get medical care, tests, or treatment you or a doctor believed necessary?” with response categories” yes”, “no”, and “don’t know.”

Having had an ED visit was collected from the question “During the past 12 months, how many times have you gone to a hospital emergency room for your own health?” with response categories of a number of visits or “don’t know.” These were made into dichotomous variables, with individuals who responded “don’t know” removed.

### Independent variable

The independent variable was whether an individual reported ever using any enabling services. Use of enabling services was defined as an individual responding yes to having received at least one enabling service from the health center from a list of questions asking about different enabling services use. Possible enabling services an individual could have reported receiving related to social determinants of health were assistance with housing, employment, child care, applying for government benefits, and obtaining food, clothes, or shoes. Individuals were also asked about enabling services related to assistance with translation, arranging medical appointments outside the health center, transportation to medical appointments, free medication assistance, health education, supportive counseling, home visits, free services outside of the health center, and assistance with other needs.

### Covariates

Covariates included in the analysis were selected a priori based on prior studies and potential hypothesized confounders [26]. Sociodemographic covariates included age, race/ethnicity, educational attainment, poverty status, language spoken at home, and type of health insurance. Age was categorized as: 18–44, 45–64, and 65+. Sex was defined as male or female. Race/ethnicity was collected and used in the analysis as: non-Hispanic White, non-Hispanic Black, Hispanic, non-Hispanic Asian, and other. Educational attainment was collected as: less than high school, high school, and more than high school. Poverty status was based on the federal poverty levels and categorized as: ≤100%, 101–138%, and ≥ 139%. Language spoken at home was categorized as: English or other language. Health insurance was categorized as: employer/union or purchased insurance, public insurance, other insurance, and not covered.

Health behavior covariates were also included in the models. Self-reported health status was categorized as: good/better versus fair/poor. Self-reported average amount of sleep per day was categorized as: at least 7 h or less than 7 h. Physical activity was categorized as: physically active at least 3 days a week or physically active less than 3 days a week. Difficulty with daily living activities was defined as any difficulty with dressing, bathing, eating, getting in or out of bed/chairs, using the bathroom, and serious difficulty walking or climbing stairs. Tobacco use was defined as: currently using tobacco or not currently using tobacco. Alcohol use was defined as: any alcohol use in the past 3 months or no alcohol use in the past 3 months. Illicit substance use was defined as: any illicit substance use in past 3 months or no illicit substance.

Additionally, the model included health condition covariates. Diabetes, hypertension, high cholesterol, anxiety, panic disorder, schizophrenia, and bipolar disorder were all defined as binary variables of whether a person had ever been told by a health professional that they had the condition. Weight problem was defined as being told by a health professional during the past 12 months they had a weight problem.

Given individual health center grant recipients can vary greatly in terms of their population served and services offered, we included CHC-level characteristics as covariates in the analysis as well as covariates for each of the 166 health centers included in the sample. Health center designation was categorized as: Community Health Center, Public Housing Primary Care, Migrant Health Center, or Health Care for the Homeless. The location of the health center, urban or rural, was also included.

### Statistical analyses

To address potential issues of selection bias we used doubly robust propensity score weighting methods with survey weights as described below, based on methods put forward by Hirano and Imbens, to create comparable groups of enabling service users and nonusers through balancing their propensity to use enabling services based on observed variables. This increases the chances that differences in outcomes between these populations is due to enabling services use and is similar to the methodology of previous national analyses of outcomes associated with enabling services use [17, 18]. Final weights were calculated through inverse probability weighting to balance the treatment and comparison group and then multiplying these weights by the survey weights included in the dataset to account for the survey design.

Covariates included in the propensity score weight development were determined by conducting logit regressions with enabling services use against every covariate individually for the overall population of adults with cardiometabolic risk factors and each subpopulation (adults with 1, 2, and ≥ 3 cardiometabolic risk factors, adults aged 18–44 and 45–64, and adults visiting rural CHCs and urban CHCs). Covariates were only included in propensity score weighting if their Z-statistic was greater than the critical value of two.

To accommodate this propensity score weighting method and ease interpretability of the findings, similar to prior research using dichotomous outcome variables [17, 27], we used linear regressions accounting for the survey sample design and incorporating the final weights to obtain the final results. To determine which covariates were included in the final linear regressions, preliminary linear regressions were run with the dependent variable being assessed against every control variable individually while controlling for enabling services use. Covariates with a t-statistic greater than two were included in the final regression adjustments [28]. For a list of the covariates included in the propensity score weighting and linear regressions, a summary of the sensitivity analyses, and the unadjusted outcomes, see Additional file 1. All analyses were conducted in Stata 15 and results were deemed statistically significant based on a two-sided *p* value of less than 0.05.

## Results

Of the 2358 respondents in the final sample, approximately 88.8% reported having received enabling services at some point from the health center. Baseline characteristics of enabling services users and nonusers with cardiometabolic risk factors before and after propensity score weighting for each variable and covariate were examined. We found that prior to propensity score weighting, the only variables with statistically significant differences between enabling service users and nonusers were health center type, high cholesterol, and recent substance use. Nonusers reported higher use of community health centers (96.0% of nonusers versus 91.0% of users) and lower use of the other types of health centers. Enabling service users had a significantly higher prevalence of high cholesterol (62.4% of users versus 46.8% of nonusers) and higher prevalence of substance use in the past 3 months (12.7% of users versus 3.1% of nonusers). After propensity score weighting the statistically significant differences in health center type and substance use were no longer present, but there were still statistically significant differences between the groups in prevalence of high cholesterol (43.4% of nonusers versus 61.4% of users). For a table of results for all of the characteristics, please see Appendix 3 in Additional file 1.

**Fig. 1:**
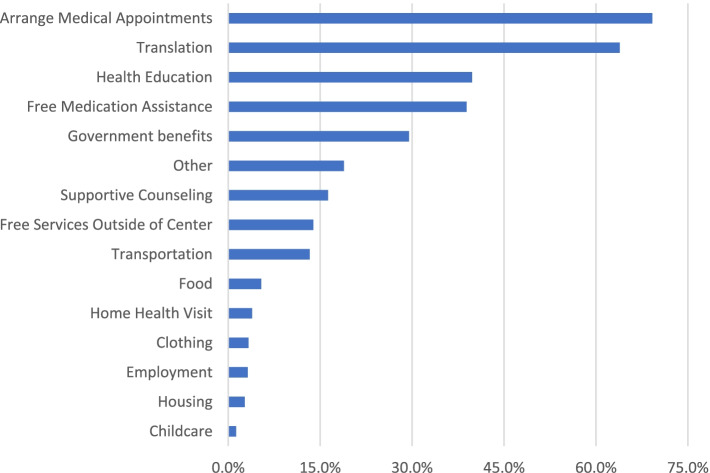
Weighted Enabling Services Used Among Adult Users with Cardiometabolic Risk Factors. Source: 2014 Health Center Patient Survey data

The distribution of type of enabling service used is presented (Fig. 1). The most common enabling services received are assistance with arranging medical appointments and translation services, with 69.2 and 63.9% of users respectively reporting having received these services. Assistance with meeting needs such as childcare and housing are the least common, with only 1.3 and 2.7% of users respectively reporting having received these services.

Table 1 shows the adjusted estimates for the outcomes examined by population. In general, the results provide support for our hypothesis that enabling services use is associated with decreases in delayed/foregone medical care (15.4 percentage points [pp]) and increases in routine check-ups (29.4 pp) among all adults with cardiometabolic risk factors, once accounting for final weights and subsequent linear regression adjustments. Statistically significant decreases in delayed/foregone care are also found for users in urban health centers (14.0 pp) and those with two risk factors (27.9 pp). There are differing decreases by age category, with a statistically significant decrease in delayed/foregone care of 54.4 pp. for individuals 18 to 44 compared to a statistically significant decrease of 24.9 pp. for individuals 45 to 64. However, the differences between age groups are not statistically significant. For routine checkups, enabling services use is associated with a statistically significant increase among adults 45–64 (39.7 pp) and a decrease in adults with three or more risk factors (7.6 pp).

**Table 1:** Adjusted outcomes associated with enabling services by population relative to individuals who did not use enabling services

	Delayed/Foregone Care (%)	Routine Check-up (%)	ED Visits (%)
Adults with Cardiometabolic Risk Factors (*N* = 2358)	−15.4*	29.4*	−15.5
# Cardiometabolic Risk Factors			
1 (*N* = 787)	2.2	−8.1	−1.4
2 (*N* = 771)	−27.9*	.3	16.1
≥ 3 (*N* = 800)	4.3	−7.6*	41.3*
Age (limited to adults with cardiometabolic risk factors)			
18–44 (*N* = 627)	− 40.6*	.7	8.4
45–64 (*N* = 1385)	−25.4*	39.7*	1.5
Geography (limited to adults with cardiometabolic risk factors)			
Rural (*N* = 761)	−24.1 ^a^	7.4^a^	− 48.5^a^
Urban (*N* = 1597)	−14.0*	1.8	−2.8

Source: Authors’ analysis of the 2014 Health Center Patient Survey data

Notes: Adjusted difference incorporates final weights and subsequent linear regression

^a^Indicates *p*-value could not be calculated because sample has a stratum with only one sampling unit

^*^p < 0.05

^**^p < 0.01

^***^p < 0.001

For ED visits, when compared to nonusers, enabling services use was associated with a statistically significant 41.3 pp. increase in visiting the ED for individuals with three or more cardiometabolic risk factors. Other subpopulations were not statistically significant and we were unable to calculate statistical significance for the 761 patients of rural health centers because the subpopulation only contains one respondent for one of the survey stratum.

## Discussion

This study finds that there are positive associations between receiving enabling services and increased receipt of timely care for individuals with cardiometabolic risk factors. Improving the timeliness of care can help to ensure that medical conditions are properly managed and do not grow into larger, more costly problems [29, 30]. These findings may in part be due to almost 70% of enabling service users reporting having received assistance arranging medical appointments and provides support for the value of care coordination. Subpopulation analyses by age and geography also found positive associations with enabling services use for one or both of these outcomes depending on the population, showing potential variation in the effectiveness of these services based on an individual’s age and location. Further study is needed to assess the value of these services for rural patients, as statistical significance could not be calculated.

This is an important issue for CHCs to address as they disproportionately treat populations with cardiometabolic risk factors [10], and while studies have generally found CHC patient health outcomes to be as good as or better than similar non-CHC patients, there is still room for improvement [31–35]. For example, CHCs do not meet the Healthy People 2030 goals for adequate control and management of diabetes [1, 36].

In contrast to the positive associations with enabling services found above, enabling services users with three or more risk factors experienced increased ED visits and decreased receipt of routine check-ups. This provides additional evidence for the increased challenges in managing multiple cardiometabolic risk factors that has been noted in previous literature [22, 23], potentially highlighting the need for increased supports beyond the enabling services provided. However, since we do not know whether the ED visits were preventable or not, we cannot say if these visits were for services enabling services could have helped to prevent. Given this gap in information, it is not possible to know if for this particularly high risk population with three or more risk factors, increased ED visits are needed and receiving this level of care is actually a positive outcome. Additionally, the decreased routine check-ups may be due to replacing these medical visits with visits to more specialized providers who can better address their health needs.

When examining the association between enabling services use and ED visits for all adults with cardiometabolic risk factors, those with fewer than three risk factors, and age and geography subgroups, there are no statistically significant associations found. While previous research has demonstrated enabling services use to be associated with decreased reports of using the ED as one’s usual source of care [18], the findings here raise questions about the association between these services and any ED use. We had hypothesized that using enabling services would be associated with decreased reports of visiting the ED in the past year. However, our null findings of the associations between enabling services use and ED visits for all populations except those with three or more risk factors highlights how ED use is more downstream than other outcomes examined and as a result can be harder for a CHC to affect than the use of services provided at the CHC such as routine check-ups. These mixed results show a need for further research as ED visits are a costly form of care, where there is room for large savings through having other providers address medical situations that do not need ED level care [37, 38].

As shown in Fig. 1, the majority of users did not receive enabling services that helped them address material needs such as employment, food, and housing. Given the link between material need and poor cardiometabolic outcomes, an emphasis on providing services that address these needs, particularly for the highest risk populations managing multiple risk factors, may help to improve outcomes. While limited, studies have shown some improvements in cardiometabolic risk factor outcomes associated with addressing housing, food, and monetary needs [16, 39, 40]. For example, Berkowitz et al. [39] found screening for resource needs and helping connect individuals with cardiometabolic risk factors to resources, such as through enrollment in benefit programs, was associated with decreases in blood pressure and cholesterol levels.

Part of the challenge in providing more intensive enabling services that could help to address needs beyond arranging medical appointments and translation, is the limited funding for these services. CHCs receive funding for enabling services mainly through HRSA Section 330 grants, Medicaid reimbursements, and other grants typically provided by state, local, or private funders. Often HRSA grants do not provide enough funding to fully cover the cost of services. State Medicaid reimbursements do not always cover enabling services and even when these services are covered, the reimbursement may not cover the full cost of care [1, 41]. This lack of full funding often leads CHCs to rely on other grants, which tend to be narrow and time-limited, inhibiting the full development and staffing of these services [41, 42]. Furthermore, CHCs may feel that enabling services are the easiest to cut when needing to reduce costs. For example, when faced with potential budget cuts due to a lapse in funding of the Community Health Center Fund (CHCF), enabling services were the top services CHCs considered reducing or eliminating [43]. To address these issues and support the provision of a variety of enabling services, improved funding for these services is needed and could be achieved through the permanent establishment of the CHCF and increased coverage of these services by state Medicaid and managed care organizations [18, 41].

The findings of this study should be interpreted in light of several strengths and limitations. This study has the strengths of being at the national level, using a survey with a high response rate, and accounting for selection bias through the use of doubly robust propensity score weighting. Additionally, this study focuses on adults with cardiometabolic risk factors, which is a particularly high priority population in the United States as these conditions account for a large number of deaths and poor health outcomes in the country [5–7]. Furthermore, since this study uses survey data, we are able to capture and account for services received outside of the CHC.

In terms of limitations, first, due to the cross-sectional nature of the data it is unknown if enabling services were received before or after individuals experienced the outcomes examined, therefore we are unable to make any conclusions about causality. Additionally, there still may be unobserved differences between enabling services users and nonusers that our analyses cannot address, further limiting causal interpretations. Next, there is a measurement issue with the limited information collected in the ED visits variable. This variable does not collect information on why the ED visit happened, so it is unknown if the outcomes are the result of something that could have been addressed by enabling services. For example, enabling services would likely not be able to prevent ED visits due to car accidents, but may be able to prevent ED visits due to poor diabetes management. Furthermore, the survey only asks if an individual has ever received certain types of enabling services, not when they received it, the frequency, or intensity. Thus, we are unable to determine if respondents received an enabling service many years ago, which would likely not affect their health care utilization in the past year. Future research on this could help to provide insight on the level of enabling services needed to have an effect on cardiometabolic health outcomes and for how long the effects of these services last. This analysis is based on self-reported data, which may be biased if an individual reports inaccurate information. Finally, at the time of this analysis the latest publicly available HCPS data was from 2014. While it is possible that there may have been changes in enabling service provision and utilization in CHCs since 2014, the findings are highly relevant because they provide compelling evidence for the adequate and stable funding of these services, which often experience underfunding and challenges with securing long-term funding [42].

## Conclusions

The value of enabling services and the importance that they be adequately funded should be emphasized through additional research to fill the gaps of what is still unknown about these services. Other studies have shown the value of enabling services for the general population [17, 18] and of certain enabling services for individuals experiencing one specific cardiometabolic risk factor [15, 42]. This is the first national level study supporting the use of these services specifically for individuals with cardiometabolic risk factors and showing the majority of enabling services provided aim to help connect individuals directly to medical care. The overall positive associations with decreasing delayed/foregone care and increasing the use of routine check-ups indicate these services may be working. This is an important finding as timely and regular care receipt can be very important for individuals with cardiometabolic risk factors to ensure their conditions stay under control. The differences in outcomes among subpopulations, highlights the importance of reporting outcomes by different population demographics and that CHCs may want to tailor enabling services to certain patient groups.

Still, future research using longitudinal data to understand the causality of enabling services on health outcomes for individuals with cardiometabolic risk factors is needed, as is further analysis on the impact of specific enabling services on health outcomes, particularly for individuals managing three or more conditions, where negative associations were found.

## Supplementary Information

**Additional file 1.** This file contains the variables included in the individual analyses, full results of the analyses, and a description of the sensitivity analyses.

## Data Availability

The dataset analyzed during the current study are available at HRSA’s Health Center Program website, https://bphc.hrsa.gov/datareporting/research/hcpsurvey/datauseragreement.html

## Abbreviations

CHC: Community Health Centers
CHCF: Community Health Center Fund
ER: Emergency Room
HRSA: Health Resources and Services Administration
PP: Percentage Points
