# Supplementary material for: Relationships between enabling services use and access to care among adults with cardiometabolic risk factors: findings from the 2014 National Health Center Patient Survey
Source: BMC Health Serv Res. 2022 Mar 14;22:334. doi: 10.1186/s12913-022-07739-3 (PMC8922912; doi:10.1186/s12913-022-07739-3)
Supplement: Supplementary file 1 — Additional file 1. This file contains the variables included in the individual analyses, full results of the analyses, and a description of the sensitivity analyses. [file 12913_2022_7739_MOESM1_ESM.docx]

**Appendix 1**

**Propensity Score Weighting Variable Selection**

The variables included in the final propensity score adjustments are described below.

**Adults with cardiometabolic risk factors:** age (45-64), mental health conditions, poor self-reported health, currently using tobacco, health insurance (other, uninsured), health center type (community health center, health care for the homeless)

**Adults with cardiometabolic risk factors at rural health centers:** race (other)

**Adults with cardiometabolic risk factors at urban health centers:** FPL (≤100%), mental health conditions, poor self-reported health, currently using tobacco, health insurance (uninsured), health center type (community health center, health care for the homeless)

**Adults 18-44 with cardiometabolic risk factors:** 7+ hours of sleep, poverty level (≥139%)

**Adults 45-64 with cardiometabolic risk factors:** mental health conditions, poor self-reported health, currently using tobacco, health insurance (uninsured), health center type (community health center, public housing primary care, health care for the homeless)

**Adults with 1 cardiometabolic risk factor:** mental health conditions, poor self-reported health, health insurance (public insurance, uninsured), health center type (community health center)

**Adults with 2 cardiometabolic risk factors:** diabetes, currently using tobacco, health center type (community health center, health care for the homeless), alcohol use in past 3 months

**Adults with 3 or more cardiometabolic risk factors:** age (18-44, 45-64), health insurance (other), health center type (health care for the homeless)

**Adults without cardiometabolic risk factors:** age (18-44, 45-64), race (non-Hispanic black), FPL (≤100%), difficulty with daily living activity**,** mental health conditions, poor self-reported health, currently using tobacco

**Appendix 2**

**Linear Regression Adjustment Variable Selection**

The variables included in the final regression adjustments are described below.

**Adults with cardiometabolic risk factors**

*Delayed/foregone care:* female, age (45-64, 65+), race (non-Hispanic black, Asian), 7+ hours of sleep, education level (less than high school, more than high school), FPL (≤100%, ≥139%), difficulty with daily living activity, weight problem in past 12 months, mental health conditions, poor self-reported health, currently using tobacco, illicit substance use in past 3 months, health insurance (employer/union or purchased insurance, uninsured), health center type (community health center, health care for the homeless)

*Emergency room visit in past 12 months:* age (18-44, 65+), race (non-Hispanic black, Asian, Hispanic), 7+ hours of sleep, education (high school), FPL (≤100%, 101-138%, ≥139%), speaks language other than English at home, difficulty with daily living activity, weight problem in past 12 months, mental health conditions, poor self-reported health, currently using tobacco, illicit substance use in past 3 months, health insurance (employer/union or purchased insurance, public insurance, other, uninsured), health center type (community health center, migrant health center, health care for the homeless)

*Routine check-up in past 12 months:* race (non-Hispanic black, Asian, Hispanic), hypertension, active at least 3 days a week, poor self-reported health, health insurance (other, uninsured), health center type (community health center, migrant health center), rural health center, alcohol use in past 3 months

**Adults with cardiometabolic risk factors at rural health centers**

*Delayed/foregone care:* 7+ hours of sleep, FPL (≤100%, ≥139%), difficulty with daily living activity, weight problem in past 12 months, mental health conditions, poor self-reported health, currently using tobacco, health insurance (employer/union or purchased insurance, uninsured), health center type (health care for the homeless)

*Emergency room visit in past 12 months:* age (65+), race (non-Hispanic black), 7+ hours of sleep, FPL (≤100%, ≥139%), speaks language other than English at home, difficulty with daily living activity, weight problem in past 12 months, mental health conditions, active at least 3 days a week, poor self-reported health, currently using tobacco, health insurance (employer/union or purchased insurance, public insurance), health center type (health care for the homeless)

*Routine check-up in past 12 months:* hypertension, active at least 3 days a week, poor self-reported health

**Adults with cardiometabolic risk factors at urban health centers**

*Delayed/foregone care:* female, age (45-64, 65+), race (non-Hispanic white, non-Hispanic black, Asian), 7+ hours of sleep, education level (less than high school, more than high school),

FPL (≤100%, ≥139%), difficulty with daily living activity, hypertension, weight problem in past 12 months, mental health conditions, poor self-reported health, health insurance (uninsured), health center type (community health center, health care for the homeless)

*Emergency room visit in past 12 months:* age (18-44, 65+), race (non-Hispanic white, non-Hispanic black, Asian, Hispanic), 7+ hours of sleep, education level (less than high school, high school), FPL (≤100%, 101-138%, ≥139%), speaks language other than English at home, difficulty with daily living activity, weight problem in past 12 months, mental health conditions, poor self-reported health, currently using tobacco, illicit substance use in past 3 months, health insurance (employer/union or purchased insurance, public insurance, other, uninsured), health center type (community health center, migrant health center, health care for the homeless)

*Routine check-up:* race (Asian, Hispanic), health insurance (uninsured), health center type (community health center, migrant health center), alcohol use in past 3 months

**Adults 18-44 with cardiometabolic risk factors**

*Delayed/foregone care:* race (non-Hispanic black, other), education level (less than high school, more than high school), FPL (≤100%), difficulty with daily living activity, high cholesterol, mental health conditions, poor self-reported health

*Emergency room visit in past 12 months:* race (non-Hispanic white, non-Hispanic black, Hispanic), 7+ hours of sleep, education level (less than high school), FPL (≤100%, 101-138%, ≥139%), speaks language other than English at home, difficulty with daily living activity, weight problem in past 12 months, mental health conditions, poor self-reported health, currently using tobacco, illicit substance use in past 3 months, health insurance (employer/union or purchased insurance, public insurance, uninsured), health center type (community health center, migrant health center, health care for the homeless)

*Routine check-up:* race (non-Hispanic, Hispanic), speaks language other than English at home, hypertension, health center type (public housing primary care, migrant health center)

**Adults 45-64 with cardiometabolic risk factors**

*Delayed/foregone care:* female, race (Asian), 7+ hours of sleep, FPL (≤100%, ≥139%), difficulty with daily living activity, weight problem in past 12 months, mental health conditions, poor self-reported health, health insurance (public insurance, uninsured), health center type (community health center, health care for the homeless)

*Emergency room visit in past 12 months:* race (non-Hispanic black, Asian, Hispanic), 7+ hours of sleep, education level (more than high school), FPL (≤100%, ≥139%), speaks language other than English at home, difficulty with daily living activity, high cholesterol, mental health conditions, poor self-reported health, currently using tobacco, illicit substance use in past 3 months, health insurance (employer/union or purchased insurance, public insurance, other, uninsured), health center type (community health center, migrant health center, health care for the homeless)

*Routine check-up:* race (Asian), 7+ hours of sleep, active at least 3 days a week, self-reported poor health, health insurance (public insurance, uninsured), rural health center, alcohol use in past 3 months

**Adults with 1 cardiometabolic risk factor**

*Delayed/foregone care:* age (45-64, 65+), race (non-Hispanic white), 7+ hours of sleep, education level (more than high school), FPL (≤100%, ≥139%), difficulty with daily living activity, mental health conditions, poor self-reported health, currently using tobacco, illicit substance use in past 3 months, health center type (health care for the homeless)

*Emergency room visit in past 12 months:* age (18-44), race (non-Hispanic white, non-Hispanic black, Asian), 7+ hours of sleep, education level (less than high school, high school education), FPL (≤100%, 101-138%, ≥139%), speaks language other than English at home, difficulty with daily living activity, weight problem in past 12 months, mental health conditions, poor self-reported health, currently using tobacco, illicit substance use in past 3 months, health insurance (public insurance, uninsured), health center type (migrant health center, health care for the homeless)

*Routine check-up:* race (non-Hispanic black, Asian, Hispanic), health center type (migrant health center), rural health center

**Adults with 2 cardiometabolic risk factors**

*Delayed/foregone care:* FPL (≤100%, ≥139%), difficulty with daily living activity, hypertension, weight problem in past 12 months, mental health conditions, poor self-reported health, health insurance (uninsured), health center type (community health center, health care for the homeless)

*Emergency room visit in past 12 months:* age (18-44, 65+), race (non-Hispanic black, Asian, Hispanic), 7+ hours of sleep, FPL (≤100%, ≥139%), speaks language other than English at home, difficulty with daily living activity, mental health conditions, poor self-reported health, currently using tobacco, illicit substance use in past 3 months, health insurance (employer/union or purchased insurance, public insurance), health center type (health care for the homeless)

*Routine check-up:* female, race (Asian, Hispanic), active at least 3 days a week, poor self-reported health, health insurance (other, uninsured), alcohol use in past 3 months

**Adults with 3 or more cardiometabolic risk factors**

*Delayed/foregone care:* female, age (18-44, 65+), race (Asian), 7+ hours of sleep, education level (less than high school, more than high school), difficulty with daily living activity, mental health conditions, poor self-reported health, health insurance (uninsured)

*Emergency room visit in past 12 months:* female, age (18-44), race (non-Hispanic black, Asian, other, Hispanic), 7+ hours of sleep, FPL (≤100%, 101-138%), speaks language other than English at home, difficulty with daily living activity, mental health conditions, poor self-reported health, currently using tobacco, illicit substance use in past 3 months, health insurance (employer/union or purchased insurance, other, uninsured), health center type (migrant health center, health care for the homeless)

*Routine check-up:* race (Asian), active at least 3 days a week, poor self-reported health, alcohol use in past 3 months

**Adults without cardiometabolic risk factors**

*Delayed/foregone care:* age (18-44, 45-64), race (other), education level (more than high school), difficulty with daily living activity, mental health conditions, poor self-reported health

*Emergency room visit in past 12 months:* race (non-Hispanic black, Asian), 7+ hours of sleep, FPL (≤100%, ≥139%), speaks language other than English at home, difficulty with daily living activity, mental health conditions, poor self-reported health, currently using tobacco, illicit substance use in past 3 months, health insurance (public insurance, uninsured), health center type (community health center, health care for the homeless)

*Routine check-up:* race (non-Hispanic black, Asian, Hispanic), FPL (101-138%), speaks language other than English at home, health insurance (public insurance, uninsured), health center type (migrant health center)

**Appendix 3**

**Adults with Cardiometabolic Risk Factors’ Characteristics/Balance Before and After Propensity Score Weighting**

|  | Survey Weights | | Final Weights | |
| --- | --- | --- | --- | --- |
| Variable | Nonusers (n=264) | Users  (n=2,094) | Nonusers (n=264) | Users (n=2,094) |
| Female | 56.5% | 59.3% | 64.5% | 59.0% |
| Race/Ethnicity^a^ |  |  |  |  |
| White Non-Hispanic | 49.7 | 54.8 | 49.8 | 54.5 |
| Black Non-Hispanic | 16.7 | 19.9 | 17.3 | 20.9 |
| Asian Non-Hispanic | 1.4 | 1.7 | 2.7 | 1.5 |
| Other Non-Hispanic | 7.6 | 4.7 | 5.2 | 4.4 |
| Hispanic or Latino | 24.6 | 18.9 | 25.0 | 18.7 |
| Federal Poverty Level^a^ |  |  |  |  |
| ≥139% Federal Poverty Level | 28.4 | 25.6 | 28.6 | 25.9 |
| 101-138% Federal Poverty Level | 10.9 | 19.1 | 12.4 | 18.1 |
| ≤100% Federal Poverty Level | 60.7 | 55.3 | 59.0 | 56.0 |
| Age in years^a^ |  |  |  |  |
| 18-44 | 42.0 | 34.3 | 35.8 | 34.3 |
| 45-64 | 49.1 | 48.7 | 55.2 | 48.1 |
| 65 and over | 8.9 | 17.04 | 9.0 | 17.6 |
| Health Insurance Status^a^ |  |  |  |  |
| Employer/Union Insurance | 18.8 | 20.7 | 18.0 | 21.0 |
| Public Insurance (Medicare, Medicaid, local safety net insurance) | 39.1 | 45.8 | 39.6 | 45.2 |
| Other Insurance | 5.6 | 9.1 | 6.1 | 8.3 |
| Not covered | 36.6 | 24.4 | 36.4 | 25.5 |
| Health Center Type^a^ |  | * |  |  |
| Community Health Center | 96.0 | 91.0 | 91.0 | 91.5 |
| Public Housing Primary Care | .4 | 1.5 | 1.0 | 1.4 |
| Migrant Health Center | 2.4 | 3.5 | 5.0 | 3.3 |
| Health Care for the Homeless | 1.2 | 4.1 | 3.2 | 3.8 |
| Rural Health Center Location | 59.9 | 55.7 | 59.8 | 56.3 |
| Cardiometabolic risk factors |  |  |  |  |
| Diabetes | 33.2 | 37.5 | 33.6 | 39.1 |
| High Cholesterol | 46.8 | 62.4* | 43.4 | 61.4* |
| Hypertension | 72.6 | 70.8 | 75.2 | 70.9 |
| Weight Problem | 46.4 | 42.9 | 43.1 | 44.2 |
| # of cardiometabolic risk factors^a^ |  |  |  |  |
| 1 risk factor | 42.2 | 32.9 | 44.8 | 32.5 |
| 2 risk factors | 26.5 | 31.6 | 24.9 | 31.0 |
| 3 risk factors | 21.5 | 24.6 | 20.7 | 25.0 |
| 4 risk factors | 9.8 | 10.9 | 9.6 | 11.5 |
| Speaks language other than English at home | 28.6 | 23.5 | 25.5 | 23.5 |
| Difficulties with any Activities of Daily Living | 27.6 | 32.2 | 32.1 | 31.3 |
| Alcohol use in past 3 months | 37.8 | 45.3 | 34.2 | 45.1 |
| Substance use in past 3 months | 3.1 | 12.7* | 6.4 | 12.6 |
| Tobacco use currently | 24.4 | 32.2 | 28.5 | 30.7 |
| Physically active at least 3 days a week | 50.9 | 63.4 | 54.8 | 63.8 |
| Mental health condition | 31.4 | 36.7 | 40.2 | 35.8 |
| Self-reported poor or fair health | 41.2 | 49.3 | 46.8 | 47.8 |
| Education level^a^ |  |  |  |  |
| Less than high school | 29.3 | 38.4 | 27.8 | 37.6 |
| High school | 31.6 | 26.3 | 32.4 | 26.0 |
| More than high school | 39.2 | 35.3 | 39.8 | 36.5 |
| Average of at least 7 hours of sleep | 47.6 | 57.8 | 44.1 | 57.6 |

Source: Authors’ analysis of the 2014 Health Center Patient Survey data.

Notes: Sample sizes based on unweighted populations. P values based on chi-square tests where *p < 0.05. ^a^One chi-square test conducted for all items in category.

**Appendix 4**

**Unadjusted and Adjusted Outcomes**

**Unadjusted and adjusted delayed/foregone care outcomes by enabling services use for all adults with cardiometabolic risk factors and by subgroup population.**

|  | Users (%) | Nonusers (%) | Unadjusted difference (%) | Adjusted difference (%) | t | R-squared (%) |
| --- | --- | --- | --- | --- | --- | --- |
| All Adults with Cardiometabolic Risk Factors (N=2,358) | 13.8% | 17.3% | -3.5% | -15.4%* | -2.71 | 46.2% |
| # Cardiometabolic Risk Factors  1 (N=787) | 9.8 | 3.3 | 6.5* | 2.2 | 0.13 | 66.8 |
| 2 (N=771) | 11.5 | 11.9 | -.4 | -27.9* | -2.53 | 61.7 |
| ≥3 (N=800) | 19.5 | 40.9 | -21.4 | 4.3 | 0.45 | 64.1 |
| Age  18-44 (N=627) | 14.5 | 28.4 | -13.9 | -40.6* | -7.18 | 75.8 |
| 45-64 (N=1,385) | 15.3 | 10.8 | 4.5 | -25.4* | -4.74 | 56.5 |
| Geography  Rural (N=761) | 12.9 | 17.9 | -5.0^a^ | -24.1 ^a^ | . ^a^ | 48.9 |
| Urban (N=1,597) | 14.8 | 16.5 | -1.7 | -14.0* | -2.21 | 52.2 |

**Source:** Authors’ analysis of the 2014 Health Center Patient Survey data.

**Notes:** Unadjusted differences only incorporate survey weights. Adjusted difference incorporates final weights and subsequent linear regression. ^a^Indicates p-value and t-statistic could not be calculated because sample has a stratum with only one sampling unit. *p < 0.05

**Unadjusted and adjusted routine check-up outcomes by enabling services use for all adults with cardiometabolic risk factors and by subgroup population.**

|  | Users (%) | Nonusers (%) | Unadjusted difference (%) | Adjusted difference (%) | t | R-squared (%) |
| --- | --- | --- | --- | --- | --- | --- |
| All Adults with Cardiometabolic Risk Factors (N=2,358) | 73.2% | 73.3% | -.1% | 29.4*% | 3.57 | 43.5% |
| # Cardiometabolic Risk Factors  1 (N=787) | 72.4 | 76.4 | -4.0 | -8.1 | -0.91 | 72.3 |
| 2 (N=771) | 73.0 | 67.9 | 5.1 | .3 | 0.02 | 62.7 |
| ≥3 (N=800) | 74.1 | 73.8 | 0.3 | -7.6* | -2.14 | 61.9 |
| Age  18-44 (N=627) | 75.1 | 71.2 | 3.9 | .7 | 0.08 | 66.2 |
| 45-64 (N=1,385) | 76.4 | 73.6 | 2.8 | 39.7* | 2.42 | 51.2 |
| Geography  Rural (N=761) | 72.4 | 71.5 | .9^a^ | 7.4^a^ | . ^a^ | 48.5 |
| Urban (N=1,597) | 74.2 | 76.0 | -1.8 | 1.8 | 0.37 | 48.4 |

**Source:** Authors’ analysis of the 2014 Health Center Patient Survey data.

**Notes:** Unadjusted differences only incorporate survey weights. Adjusted difference incorporates final weights and subsequent linear regression. ^a^Indicates p-value and t-statistic could not be calculated because sample has a stratum with only one sampling unit. *p < 0.05

**Unadjusted and adjusted ER visit outcomes by enabling services use for all adults with cardiometabolic risk factors and by subgroup population.**

|  | Users (%) | Nonusers (%) | Unadjusted difference (%) | Adjusted difference (%) | t | R-squared (%) |
| --- | --- | --- | --- | --- | --- | --- |
| All Adults with Cardiometabolic Risk Factors (N=2,358) | 50.7% | 37.6% | 13.1%* | -15.5 | -1.29 | 53.0% |
| # Cardiometabolic Risk Factors  1 (N=787) | 43.1 | 36.8 | 6.3 | -1.4 | -0.04 | 75.5 |
| 2 (N=771) | 47.8 | 33.8 | 14 | 16.1 | 0.71 | 78.1 |
| ≥3 (N=800) | 60.5 | 42.0 | 18.5 | 41.3* | 2.42 | 66.7 |
| Age  18-44 (N=627) | 60.0 | 57.6 | 2.4 | 8.4 | 0.30 | 76.6 |
| 45-64 (N=1,385) | 47.3 | 23.4 | 23.9* | 1.5 | 0.09 | 59.3 |
| Geography  Rural (N=761) | 53.0 | 43.0 | -10.0^a^ | -48.5^a^ | .^a^ | 59.1 |
| Urban (N=1,597) | 47.9 | 29.6 | 18.3* | -2.8 | -0.36 | 58.0 |

**Source:** Authors’ analysis of the 2014 Health Center Patient Survey data.

**Notes:** Unadjusted differences only incorporate survey weights. Adjusted difference incorporates final weights and subsequent linear regression. ^a^Indicates p-value and t-statistic could not be calculated because sample has a stratum with only one sampling unit. *p < 0.05

**Appendix 5**

**Sensitivity Analyses**

Multiple sensitivity analyses were conducted to test the robustness of the results. First, due to the initial survey sample weights being large, with the largest initial weight being approximately 52,266, when these weights were multiplied by the propensity score weights, they became even larger. To address this issue weight trimming at the 95^th^ percentile was tested.[1] This resulted in the range of weights being even smaller than the range of the initial survey weights, but did not have much of an effect on the results (shown in Exhibit 1). Therefore, since the large weights did not seem to overly influence results, the full weights were used.

To further assess the impact of the weights, analyses using no weights and only propensity score weights without multiplying by the survey design weights were conducted. Results for all of these sensitivity analyses are shown below in Exhibit 1, and demonstrate that while the level of significance and magnitude for the coefficients vary, the general finding that enabling services use is associated with decreased delayed/foregone care and increased routine check-ups for adults with cardiometabolic risk factors remains the same. Though results vary for ER visits, we chose to continue with the full weights to account for the survey design and potential issues of selection bias.

**Exhibit 1. Weight Sensitivity Analyses**

| Adults with Cardiometabolic Risk Factors | Delayed/Foregone Care (%) | Routine Check-up (%) | Any ER Visit (%) |
| --- | --- | --- | --- |
| Full Weights (N=2,358) | -15.4%* | 29.4%* | -15.5% |
| Weights Trimmed at 95^th^ Percentile (N=2,358) | -15.6* | 30.8* | -6.8 |
| Propensity Score Weights Only (N=1,836) | -18.2* | 26.9* | -14.4* |
| No Weights (N=2,358) | -12.6* | 21.1* | -13.4* |

*p< 0.05

Comparing these results to adults without cardiometabolic risk factors was also tested to see if outcomes were specific to our population of focus. This changed the sample size to 742 individuals. Exhibit 2 shows that results are only statistically significant for enabling services use being associated with an increase in routine check-ups for this population. However, since the population of focus for this study was adults with cardiometabolic risk factors, we did not include these results in the main text.

**Exhibit 2. Adults without Cardiometabolic Risk Factors Sensitivity Analysis**

|  | Delayed/Foregone Care (%) | Routine Check-up (%) | Any ER Visit (%) |
| --- | --- | --- | --- |
| Adults without Cardiometabolic Risk Factors (N=742) | 3.3% | 17.4%* | -24.0% |

*p < 0.05

Finally, to decrease the number of control variables in the analyses and potentially allow for analyses with smaller populations, we tested removing the covariates for each of the 166 health center grantees from the regression adjustment. As shown in Exhibit 3, this resulted in outcomes opposite to our initial analysis with enabling services use being statistically significantly associated with an increase in the likelihood of having an ER visit in the past 12 months. While the coefficients on delayed/foregone care and routine check-ups are not statistically significant. This provides justification for including all of the health center grantee covariates, as there are likely large differences between health centers such as in their quality of care, types of services they provide, and populations they serve that need to be controlled for in the analyses. Thus, these sensitivity analyses helped to provide support for our methodology of using the full weights and including the health center grantee covariates.

**Exhibit 3. Removing Health Center Grantee Covariates Sensitivity Analyses**

|  | Delayed/Foregone Care (%) | Routine Check-up (%) | Any ER Visit (%) |
| --- | --- | --- | --- |
| Adults with Cardiometabolic Risk Factors (N=2,358) | 2.3% | 1.2% | 10.1%* |

*p < 0.05

**References**

1. Lee BK, Lessler J, Stuart EA. Weight Trimming and Propensity Score Weighting. PLoS ONE. 2011;6(3):e18174.
